# *TLR7* rs179008 (A/T) and *TLR7* rs3853839 (C/G) polymorphisms are associated with variations in IFN-α levels in HTLV-1 infection

**DOI:** 10.3389/fimmu.2024.1462352

**Published:** 2024-11-22

**Authors:** Emmanuelle Giuliana Mendes Santana, Fabiane dos Santos Ferreira, Wandrey Roberto dos Santos Brito, Felipe Teixeira Lopes, Aline Cecy Rocha de Lima, Gabriel dos Santos Pereira Neto, Ednelza da Silva Graça Amoras, Sandra Souza Lima, Carlos Araujo da Costa, Maísa Silva Souza, Ricardo Ishak, Izaura Maria Vieira Cayres-Vallinoto, Antonio Carlos Rosário Vallinoto, Maria Alice Freitas Queiroz

**Affiliations:** ^1^ Laboratory of Virology, Institute of Biological Sciences, Federal University of Pará, Belém, Brazil; ^2^ Laboratory of Cellular and Molecular Biology, Tropical Medicine Center, Federal University of Pará, Belém, Brazil

**Keywords:** HTLV-I infection, toll-like receptor 7, polymorphism genetic, gene expression profile, interferon-alpha

## Abstract

**Introduction:**

TLR7 detects the presence of single-stranded RNA (ssRNA) viruses, including human T-lymphotropic virus 1 (HTLV-1), and triggers antiviral and inflammatory responses that are responsible for infection control. Genetic variations in the *TLR7* gene may alter cytokine production and influence the course of HTLV-1 infection. In the present study, the associations of *TLR7* gene polymorphisms with HTLV-1-related symptoms, receptor expression levels, IFN-α and TNF-α levels and the proviral load were investigated.

**Methods:**

Blood samples from 159 individuals with HTLV-1 infection (66 with inflammatory diseases and 93 asymptomatic individuals) and 159 controls were collected. The genotyping of polymorphisms, TLR7 gene expression analysis and the quantification of the proviral load were performed by real-time PCR, and cytokine measurement was performed by enzyme-linked immunosorbent assay (ELISA).

**Results:**

Carriers of the polymorphic allele for *TLR7* rs179008 (A/T) had lower levels of IFN-α, while carriers of the polymorphic allele for *TLR7* rs3853839 (C/G) had higher levels of TLR7 and IFN-α expression. The polymorphisms were not associated with symptoms of diseases related to HTLV-1 infection. The combination of A/G alleles for the *TLR7* rs179008 (A/T) and *TLR7* rs3853839 (C/G) polymorphisms was associated with increased IFN-α levels and a decreased proviral load.

**Discussion:**

Although the polymorphisms did not influence the presence of symptoms of diseases caused by HTLV-1, carriers of the wild-type alleles for *TLR7* rs179008 (A/T) and the polymorphism for *TLR7* rs3853839 (C/G) appears to have a stronger antiviral response and increased infection control.

## Introduction

Human T-lymphotropic virus 1 (HTLV-1) is the causative agent of adult T-cell leukemia (ATL) and several types of inflammatory conditions, including HTLV-1-associated myelopathy (HAM), uveitis, infectious dermatitis, rheumatoid arthritis, bronchitis and bronchiectasis (1, 2). It is estimated that 5 to 10 million people worldwide are infected with the virus (3), approximately 5% of whom may develop some of the diseases and conditions associated with HTLV-1 (4).

The factors responsible for triggering the manifestations of HTLV-1-associated diseases are not fully understood. Therefore, several studies have investigated the association of inflammatory molecular markers, especially those for HAM, with susceptibility to infection and the presence of diseases and have shown that variations in the expression of these markers may contribute to the pathogenesis of HTLV-1-infection-associated diseases (5, 6). Genetic variations in components of immunity have been associated with changes in the inflammatory response, susceptibility to infection and the presence of HTLV-1-related diseases (7–9).

Toll-like receptors (TLRs) recognize pathogen-associated molecular patterns (PAMPs) in innate immunity and play essential roles in the immune response against viral infection (10–12). TLR7 is located in the endosome and recognizes single-stranded RNA (ssRNA) of several viruses, such as HTLV. The activation of TLR7 triggers a signaling cascade, inducing the production of type I interferons (IFN-α and IFN-β), which are responsible for suppressing viral replication and inflammatory cytokines (10, 13, 14). The role of IFN-α in HTLV-1 infection was evaluated in an experimental study in different cell lines, which demonstrated that IFN-α suppresses the expression of HTLV-1 genes, resulting in the inhibition of p19 and the HTLV-1 tax protein (15), although the virus is also capable of reducing IFN-I signaling, evading the immune response (16). Another host cell mechanism against viral infections is the inflammatory response, mediated by the production of cytokines (17). However, in HTLV-1 infection, the marked increase in inflammatory cytokines, mainly TNF-α and IFN-γ, can induce the development of diseases (18, 19).

The *TLR7* gene is located on the X chromosome and has several polymorphisms potentially associated with human diseases (20–22). The *TLR7* rs179008 (A/T) single nucleotide polymorphism (SNP) promotes an amino acid change, glutamine to leucine (Gln11Leu), in the protein structure of the receptor. This change alters receptor functionality and intracellular signaling and has been linked to several viral infections, including human immunodeficiency virus 1 (HIV-1), hepatitis C virus (HCV) and severe acute respiratory syndrome coronavirus 2 (SARS-CoV-2) (12, 23–26).

The *TLR7* rs3853839 SNP (C/G) is located in the intergenic noncoding region (3’ UTR) and has been associated with different types of diseases. This genetic variant may have a substantial impact on gene expression, as the 3’ UTR is an essential regulatory region, regulating the translation, degradation and subcellular localization of mRNAs (27–29). The *TLR7* rs3853839 (G/C) variation was found to be associated with the severity of coronavirus disease 2019 (COVID-19) (30) and with the persistence of HCV infection (29).

Because information on the role of TLR7 in HTLV-1 infection is limited, and there is lack of studies on the influence of genetic variations in the *TLR7* gene on infection, in the present study, the associations of *TLR7* rs179008 (A/T) and *TLR7* rs3853839 (C/G) with susceptibility to HTLV-1 infections, the presence of symptoms of inflammatory diseases associated with infection, *TLR7* gene expression, IFN-α, inflammatory cytokines and the proviral load were investigated.

## Materials and methods

### Study population and sample collection

The present study included 159 blood samples from individuals living with HTLV-1 infection, comprising 66 patients with a clinical diagnosis of inflammatory diseases (HAM, n=46; rheumatological manifestations, n=18; dermatitis, n=1; and uveitis, n=1) and 93 asymptomatic patients. All individuals who presented a clinical diagnosis of inflammatory disease were defined as symptomatic. Individuals of both sexes, aged over 18 years, without treatment with glucocorticoids and treated at the outpatient clinic of the Tropical Medicine Center and Care Service for People Living with HTLV (SAPEVH), both a part of the Federal University of Pará, were included. Individuals with co-infection and autoimmune disease were excluded from the study.

A 10 mL sample of blood was collected by intravenous puncture into a vacuum collecting tube containing ethylenediaminetetraacetic acid (EDTA) as an anticoagulant. The samples were centrifuged and separated into aliquots of plasma and leukocytes. The leukocyte samples were used for genomic DNA extraction to *TLR7* SNPs (rs179008 and rs3775291) genotyping and quantify the proviral load. Plasma samples were used for the evaluation of plasma cytokine levels.

The control group consisted of 159 blood samples from individuals negative for infection by HTLV-1/2, HIV-1/2, HCV and hepatitis B virus (HBV), over 18 years of age and without diagnosis of an autoimmune disease. These control samples were used to compare the frequencies of polymorphisms in HTLV-1 samples. The control group was matched in age and sex with a group of people with HTLV-1. As individuals in the control group were from the same population as individuals in the HTLV-1 group; therefore, there were no interethnic differences between groups.

### DNA extraction

DNA was extracted from leukocytes from whole blood using a Puregene™ kit (Gentra Systems, Inc., Minneapolis, Minnesota, USA) following the manufacturer’s protocol, which consisted of cell lysis, protein precipitation and precipitation and hydration of the DNA. After extraction, the DNA obtained was quantified by spectrophotometric reading on a BioDrop™ instrument (Bio-Rad, Hercules, California, USA) following the protocol recommended by the manufacturer.

### Genotyping of *TLR7* rs179008 (A/T) and *TLR7* rs3853839 (C/G)

The genotypes of the investigated polymorphisms were identified by real-time PCR using the StepOnePLUS™ Real-Time PCR System (Thermo Fisher, Carlsbad, California, USA). The reactions consisted of the following commercially obtained TaqMan™ assays: *TLR7* rs179008 (C:_2259574_10) and *TLR7* rs3853839 (C:_2259573_10), with primers and probes specific for amplification of the target sequence (Thermo Fisher, Carlsbad, California, USA). The reaction consisted of 1X MasterMix, H_2_O, 20X assay C_11537906_20 and 50 ng of DNA. The following cycling conditions were used: 10 minutes at 95°C and 40 cycles of 15 seconds at 95°C and 1 minute at 60°C.

### RNA extraction

Total RNA was extracted from peripheral blood leukocytes using the TRIzol™ Plus RNA Purification Kit (Thermo Fisher Scientific, Waltham, Massachusetts, USA). The steps followed the protocol recommended by the manufacturer. The concentration of the extracted RNA was determined using a BioDrop™ (Bio-Rad, Hercules, California, USA) according to the manufacturer’s instructions. All total RNA samples had concentrations equal to 50 ng/µL for the synthesis of complementary DNA (cDNA).

### Reverse transcription

The synthesis of cDNA from RNA was performed using the High-Capacity cDNA Reverse Transcription^®^ with RNAse Inhibitor Kit (Applied Biosystems, Foster City, CA, USA). For each reaction, a mixture with a final volume of 20.0 µL was prepared, contained 2 µL of 10X RT Buffer, 0.8 µL of 25X dNTP Mix (100 nM), 2 µL of random primer, 1 µL of MultiScribe™ Reverse Transcriptase, 1 µL of RNaseOUT™ and 3.2 µL of ultrapure water supplied in the kit and 10.0 µL of extracted RNA. Subsequently, the mixture was placed in a Mastercycler personal thermal cycler (Eppendorf, Hamburg, Germany) and subjected to cycles of 25°C for 10 minutes, 37°C for 120 minutes and 85°C for 5 minutes.

### Quantification of gene expression

Gene expression was performed by quantifying mRNA by real-time PCR. First, the standardization of the quantitative PCRs (qPCRs) with the cDNAs and probes (endogenous and target genes) was performed to calculate the efficiency of the amplification reactions. In the standardization reactions, different concentrations of cDNA were tested (pure and in 4 dilutions with a factor of 2—1:2, 1:4, 1:8 and 1:16). All reactions were performed on plates and in triplicate, and the same cDNA (at different dilutions) was analyzed with different probes to construct an efficiency curve to validate the 2^-ΔΔCT^ analysis method. All tests showed efficiency as expected (100% ± 10) (31).

The relative quantification of gene expression consisted of the amplification of the target gene with the endogenous gene (normalizer) using TaqMan™ assays (Applied Biosystems, Foster City, CA, USA) and the StepOnePLUS™ Real-Time PCR System (Thermo Fisher Scientific, Waltham, MA, USA). The reactions were performed in the singleplex format according to the manufacturer’s protocol. The assay used for *TLR7* was Hs01933259_s1, and glyceraldehyde-3-phosphate dehydrogenase (GAPDH) was used as the endogenous control (Hs02786624_g1). All assays were obtained commercially (Thermo Fisher Scientific, Waltham, MA, USA). For the reaction, 5 µL of 2X TaqMan^®^ Universal PCR Master Mix, 0.5 µL of 20X TaqMan Gene Expression Assay, 1 µL of cDNA and 10.5 µL of RNAse-free water were used, along with the following thermocycling conditions: 2 minutes at 50°C, followed by 10 minutes at 95°C and 1 minute at 60°C.

The relative quantification (RQ) of target gene expression was determined using the comparative CT method (ΔΔCt) with the formula 2-ΔΔCT, where ΔΔCt = ΔCt sample- ΔCt reference (Life Technologies, Carlsbad, CA, USA).

### Plasma measurement of cytokine levels

The plasma levels of the cytokines IFN-α and TNF-α were measured using the Ready-SET-Go^®^ enzyme-linked immunosorbent assay (ELISA) (eBioscience, California, San Diego, USA), which uses specific monoclonal antibodies to detect each cytokine and measure its level. The test was performed according to the manufacturer’s instructions.

### Quantification of the HTLV-1 proviral load

The proviral load was quantified by qPCR using a TaqMan^®^ system (Applied Biosystems, Foster City, CA) with two target sequences (the albumin gene as an endogenous control and the nonhomologous region of the HTLV-1 pol gene), according to a previously described protocol (32), which starts with the extraction of DNA from leukocytes, followed by relative quantification via real-time PCR, using reaction-specific primers and probes: HTLV-1-F: GAACGCTCTAATGGCATTCTTAAAACC, HTLV-1-R: GTGGTTGATTGTCCATAGGGCTAT and HTLV-1-Probe: FAM-ACAAACCCGACCTACCC-NFQ for HTLV-1; ALB-F: GCTCAACTCCCTATTGCTATCACA, ALB-R: GGGCATGACAGGTTTTGCAATATTA and ALB-Probe: FAM-TTGTGGGCTGTAATCAT-NFQ for endogenous control (albumin). The result is further adjusted to an absolute proviral quantification considering the leukocyte count per mm^3^, and the result is expressed as the number of proviral DNA copies/mm^3^.

### Statistical analysis

The genotypic and allelic frequencies of the polymorphisms were estimated by direct counting, and the significance of the differences between the studied groups was calculated using the chi-square (χ2) test, Fisher’s exact test and the G test. The choice of tests was based on the assumptions of sample size and number of categories, as already established (33). The Hardy-Weinberg equilibrium calculation was performed to evaluate whether the observed genotypic frequency distributions were in accordance with expectations. Analyses of the normality of the distribution of *TLR7* expression levels, the proviral load and cytokine levels were performed using the Shapiro−Wilk test. The *TLR7* expression levels, proviral load levels and cytokine levels among the investigated groups were evaluated using the nonparametric Mann−Whitney test. The multiple logistic regression test evaluated the associations of the investigated variables with the presence of disease symptoms. Multiple linear regression analysis was performed to evaluate the association between the gene expression of TLR7, INF-α and TNF-α in relation to polymorphic alleles of *TLR7* rs17008, polymorphic alleles of *TLR7* rs3853839 and the proviral load of the virus. All tests were performed using BioEstat 5.3 and GraphPad Prism 5.0 software, those with a value of *p ≤* 0.05 were considered significant associations.

### Ethical aspects

This study was submitted to and approved by the Ethics Committee for Research with Human Beings of the Institute of Health Sciences of the Federal University of Pará (CAAE no. 63427822.3.0000.0018). All individuals included in the study signed an informed consent form.

## Results

### Frequency of *TLR7* rs179008 (A/T) and *TLR7* rs3853839 (C/G) polymorphisms

Of the 159 individuals living with HTLV-1 included in the study, 111 (69.81%) were female and 48 were male (30.19%), with a mean age of 49.95 years (19-80 years). The majority of individuals in the asymptomatic group (66.7%; n=62) and symptomatic (74.2%; n= 49) were female (*p*= 0.3812). The mean age of the asymptomatic group was 48 years and the symptomatic group was 51.1 years (*p*= 0.2146). The samples from the control group were paired for sex and age with those from the HTLV-1 group. Because the *TLR7* gene is located on the X chromosome, comparisons of the genotypic frequencies of the *TLR7* rs179008 (A/T) and *TLR7* rs3853839 (C/G) polymorphisms between the investigated groups were initially performed according to sex. Next, an evaluation was performed, regardless of sex, of the frequency of alleles related with (allele T) and without (allele A) alteration in the structure of the *TLR7* protein for the rs179008 (A/T) polymorphism and with (allele G) and without (allele C) alterations in the expression levels of the *TLR7* gene for the rs3853839 (C/G) polymorphism. The comparison between the frequency of genotypes for the *TLR7* rs179008 A/T polymorphism between the control and HTLV-1 groups did not show significant differences according to sex, although the frequency of the polymorphic allele (T) was greater in females in the HTLV-1 group, with a *p* value close to significance. The genotypic frequencies of the *TLR7* rs3853839 variation (C/G) did not differ between the groups in both sexes (**Table 1**).

**Table 1:** Comparison of the genotypic frequencies of the investigated polymorphisms in the *TLR7* gene between the control and HTLV-1 groups according to sex.

Genotypic profile	Control	HTLV-1	*p*
	n= 159	n= 159	
	n (%)	n (%)	
*TLR7* rs179008 (A/T)			
	Female		
	n= 111	n= 111	
AA	83 (74.77)	71 (63.96)	0.1246^a^
AT	24 (21.63)	30 (27.03)	
TT	4 (3.60)	10 (9.01)	
	Male		
	n= 48	n= 48	
A	43 (89.58)	43 (89.58)	1.0000^c^
T	5 (10.42)	5 (10.42)	
*TLR7* rs3853839 (C/G)			
	Female		
	n= 111	n= 111	
CC	45 (40.54)	39 (35.14)	0.7081^a^
CG	46 (41.44)	50 (45.05)	
GG	20 (18.01)	22 (19.81)	
	Male		
	n= 48	n= 48	
C	36 (75.00)	33 (68.75)	0.6498^c^
G	12 (25.00)	15 (31.25)	

n, number of individuals; ^a^G test; ^c^Fisher's exact test.

The analysis of genotypic frequencies for *TLR7* rs179008 (A/T) showed that female individuals with symptoms of HTLV-1-associated diseases had a greater frequency of polymorphic genotypes (AT and TT) than individuals without symptoms (*p*= 0.0260). The frequency of the polymorphic allele (T) was greater in the symptomatic (*OR*= 2.32; *p*= 0.0250). The symptomatic group had a greater frequency of carriers of the homozygous and heterozygous (AT+TT) polymorphic genotypes than the symptomatic group (*OR* = 3.00; *p* = 0.0019). Among male individuals, there was no significant difference in the frequency of genotypes for the *TLR7* rs179008 (A/T) polymorphism between the groups with and without disease symptoms. The comparison of the genotype frequencies of the *TLR7* rs3853839 (C/G) variation was not different between the groups in both sexes (**Table 2**).

**Table 2:** Comparison of the genotypic frequencies of the investigated polymorphisms in the *TLR7* gene between asymptomatic individuals and those with symptoms of HTLV-1-associated diseases according to sex.

Genotypic profile	Asymptomatic	Symptomatic	*p*	*OR* (95% CI)
	n=93	n=66		
	n (%)	n (%)		
*TLR7* rs179008 (A/T)				
	Female			
	n= 62	n= 49		
AA	46 (75.81)	25 (51.02)	0.0350^a^	-
AT	11 (17.74)	19 (38.78)		
TT	5 (6.45)	5 (10.20)		
AT+TT ^∞^	16 (24.19)	24 (48.98)	0.0200^a^	2.76 (1.24-6.13)
	Male			
	n=31	n= 17		
A	29 (93.55)	14 (82.35)	0.3306^c^	-
T	2 (6.45)	3 (17.65)		
*TLR7* rs3853839 (C/G)				
	Female			
	n= 62	n= 49		
CC	24 (38.71)	15 (30.61)	0.6419^b^	
CG	27 (43.55)	23 (46.94)		-
GG	11 (17.74)	11 (22.45)		
CG+GG ^Δ^	38 (61.29)	34 (69.39)	0.5953^b^	-
	Male			
	n=31	n= 17		
C	22 (70.97)	11 (64.71)	0.9028^c^	-
G	9 (29.03)	6 (35.29)		

n, number of individuals; ^a^G test; ^b^chi-square test; ^c^ Fisher's exact test; ^∞^AT+TT *vs*. AA; ^Δ^CG+GG *vs*. CC. *OR*, *odds ratio*; CI, confidence interval.

The frequency of individuals carrying the *TLR7* rs179008 (A/T) and *TLR7* rs3853839 (C/G) polymorphisms was also evaluated regardless of sex. For the *TLR7* rs179008 (A/T) polymorphism, individuals were classified as carriers of the A allele, including the AA (female) and A (male) genotypes; the A allele is related to the maintenance of the TLR7 receptor structure. Carriers of the T allele included individuals with AT and TT (feminine) and T (male) genotypes; the T allele is related to changes in TLR7 protein structure. For the *TLR7* rs3853839 (C/G) variation, individuals were classified as carriers of the C allele, including the CC (female) and C (male) genotypes; the C allele is unrelated to alterations in *TLR7* gene expression. Carriers of the G allele included those with the CG and GG (female) and G (male) genotypes; the G allele is associated with increased *TLR7* expression levels.

The evaluation of the frequencies of carriers with T and A allele for the *TLR7* rs179008 (A/T) polymorphism did not significantly differ between the control group and the HTLV-1 group. A similar result was observed when comparing the frequencies of carriers of the G and C allele for the *TLR7* rs3853839 (C/G) polymorphism between the investigated groups (**Table 3**).

**Table 3:** Frequency of carriers of alleles related to alteration in protein structure for the *TLR7* rs179008 (A/T) polymorphism and carriers of alleles related to changes in expression levels for *TLR7* rs3853839 (C/G) between the control and HTLV-1 groups.

Alleles	Control	HTLV-1	p*
	n= 159	n= 159	
	n (%)	n (%)	
*TLR7* rs179008 (A/T)			
A (unchanged structure of TLR7 protein)	126 (72.33)	114 (79.25)	0.1514
T (altered structure of TLR7 protein)	33 (27.67)	45 (20.75)	
*TLR7* rs3853839 (C/G)			
C (unchanged *TLR7* expression)	72 (45.28)	81 (50.94)	0.9175
G (increased expression de *TLR7*)	87 (54.71)	98 (49.06)	

n, number of individuals; * Chi-square test. Allele A: includes carriers of the AA (female) and A (male) genotypes; allele T: includes carriers of the AT and TT (female) and T (male) genotypes; allele C: includes carriers of the CC (female) and C (male) genotypes; Allele G: includes carriers of the CG and GG (female) and G (male) genotypes.

According to the presence and absence of symptoms associated with HTLV-1 infection, the frequency of carriers of alleles related to structural alterations in the protein (T) for the *TLR7* rs179008 (A/T) polymorphism was greater in the symptomatic group (*OR*= 3.10; *p*= 0.0030). The frequencies of carriers of alleles related (G) and not related (C) to changes in *TLR7* expression levels for the *TLR7* rs3853839 variation (C/G) were not significantly different between groups (**Table 4**).

**Table 4:** Frequency of carriers of alleles related to alteration in protein structure for the *TLR7* rs179008 (A/T) polymorphism and carriers of alleles related to changes in expression levels for *TLR7* rs3853839 (C/G) between asymptomatic individuals and those with symptoms of HTLV-1-associated diseases.

Alleles	Asymptomatic	Symptomatic	p*	*OR* (95% CI)
	n=93	n=66		
	n (%)	n (%)		
*TLR7* rs179008 (A/T)				
A (unchanged structure of TLR7 protein)	75 (81.72)	39 (59.09)	0.0060	2.88 (1.41-5.87)
T (altered structure of TLR7 protein)	18 (18.28)	27 (40.91)		
*TLR7* rs3853839 (C/G)				
C (unchanged *TLR7* expression)	46 (49.46)	26 (39.39)	0.2735	-
G (increased expression de *TLR7*)	47 (50.54)	40 (60.61)		

n, number of individuals; * Chi-square test. *OR*, *odds ratio*; CI, confidence interval. Allele A: includes carriers of the AA (female) and A (male) genotypes; allele T: includes carriers of the AT and TT (female) and T (male) genotypes; allele C: includes carriers of the CC (female) and C (male) genotypes; Allele G: includes carriers of the CG and GG (female) and G (male) genotypes.

### *TLR7* gene expression levels

**Figure 1:**
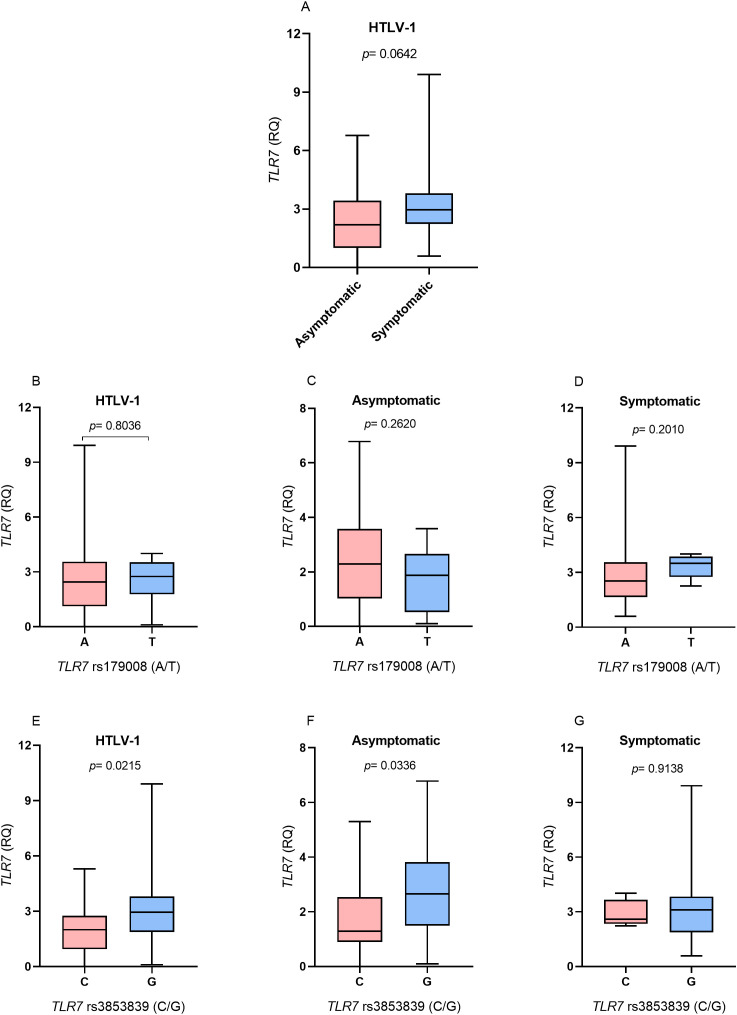
Evaluation of *TLR7* gene expression levels between **(A)** Asymptomatic individuals and those with symptoms of HTLV-1-associated diseases; **(B)** Individuals living with HTLV-1; **(C)** asymptomatic individuals and **(D)** symptomatic individuals carrying the alleles related and not related to alterations in the protein structure for the *TLR7* rs179008 (A/T) polymorphism. **(E)** Individuals living with HTLV-1, **(F)** asymptomatic individuals and **(G)** symptomatic individuals carrying the alleles related and not related to changes in expression levels for *TLR7* rs3853839 (C/G). RQ, relative quantification. Mann-Whitney test. Allele A: includes carriers of the AA (female) and A (male) genotypes; allele T: includes carriers of the AT and TT (female) and T (male) genotypes; allele C: includes carriers of the CC (female) and C (male) genotypes; Allele G: includes carriers of the CG and GG (female) and G (male) genotypes.

*TLR7* gene expression levels were higher in the symptomatic group than in the asymptomatic group, but there was no statistically significant difference (*p*= 0.0642; **Figure 1A**). In the evaluation of *TLR7* expression levels in relation to the *TLR7* rs179008 (A/T) polymorphism, no differences were observed in the levels of *TLR7* expression between individuals carrying the alleles related (T allele) and not related (A allele) to the alteration of the protein structure of TLR7 in individuals with HTLV-1 (**Figure 1B**). There were also no differences in the levels of *TLR7* expression between carriers of the A and T alleles according to the absence (**Figure 1C**) or presence (**Figure 1D**) of symptoms associated with HTLV-1 infection.

The evaluation of *TLR7* expression levels in relation to the *TLR7* rs3853839 polymorphism (C/G) revealed that individuals who were HTLV-1 carriers of the allele related to changes in receptor expression levels (G) had higher *TLR7* expression levels (*p*= 0.0215; **Figure 1E**). According to the analysis of the presence and absence of symptoms of diseases associated with HTLV-1, in the asymptomatic group, individuals carrying the G allele had higher levels of *TLR7* gene expression (*p*= 0.0336; **Figure 1F**). In contrast, no differences in the levels of *TLR7* expression were observed between individuals carrying the C and G alleles in the symptomatic group (**Figure 1G**).

### IFN-α concentration

**Figure 2:**
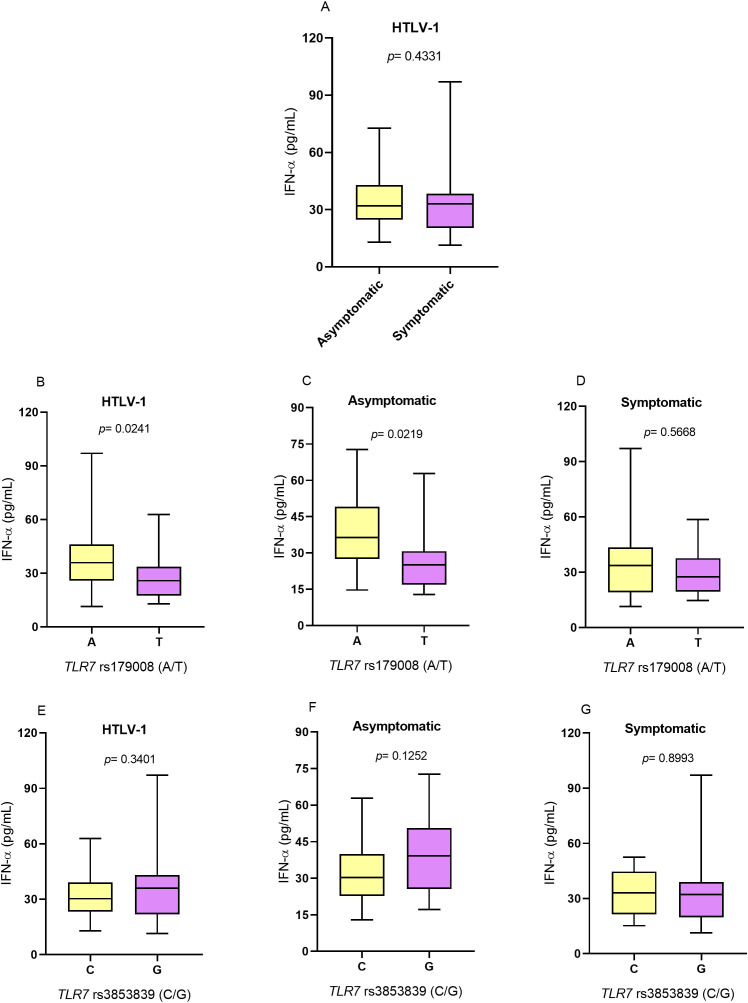
Evaluation of IFN-α levels. **(A)** Asymptomatic individuals and those with symptoms of HTLV-1-associated diseases. **(B)** Individuals living with HTLV-1, **(C)** asymptomatic individuals and **(D)** symptomatic individuals carrying the alleles related and not related to alterations in the protein structure for the *TLR7* rs179008 (A/T) polymorphism. **(E)** Individuals living with HTLV-1, **(F)** asymptomatic individuals and **(G)** symptomatic individuals carrying the alleles related and not related to changes in expression levels for *TLR7* rs3853839 (C/G). Mann-Whitney test. Allele A: includes carriers of the AA (female) and A (male) genotypes; allele T: includes carriers of the AT and TT (female) and T (male) genotypes; allele C: includes carriers of the CC (female) and C (male) genotypes; Allele G: includes carriers of the CG and GG (female) and G (male) genotypes.

IFN-α levels did not differ between individuals with and without symptoms associated with HTLV-1 infection (**Figure 2A**). Regarding the *TLR7* rs179008 (A/T) polymorphism, individuals with HTLV-1 carrying the allele related to alterations in the structure of the TLR7 protein (T allele) had lower levels of IFN-α than individuals carrying the allele not related to alteration (A allele) (*p*= 0.0241; **Figure 2B**). In the group of asymptomatic individuals, carriers of the T allele also had lower IFN-α levels than carriers of the A allele (*p*= 0.0219; **Figure 2C**). However, there were no differences in IFN-α levels between individuals carrying alleles related and not related to changes in the structure of the TLR7 protein in the symptomatic group (**Figure 2D**).

For the *TLR7* rs3853839 (C/G) polymorphism, there were no significant differences in cytokine levels between individuals carrying the alleles related (G) and not related (C) to alterations in the levels of *TLR7* expression in individuals with HTLV-1 (**Figure 2E**) and according to the absence (**Figure 2F**) and presence (**Figure 2G**) of symptoms of HTLV-1-associated diseases.

### TNF-α concentration

**Figure 3:**
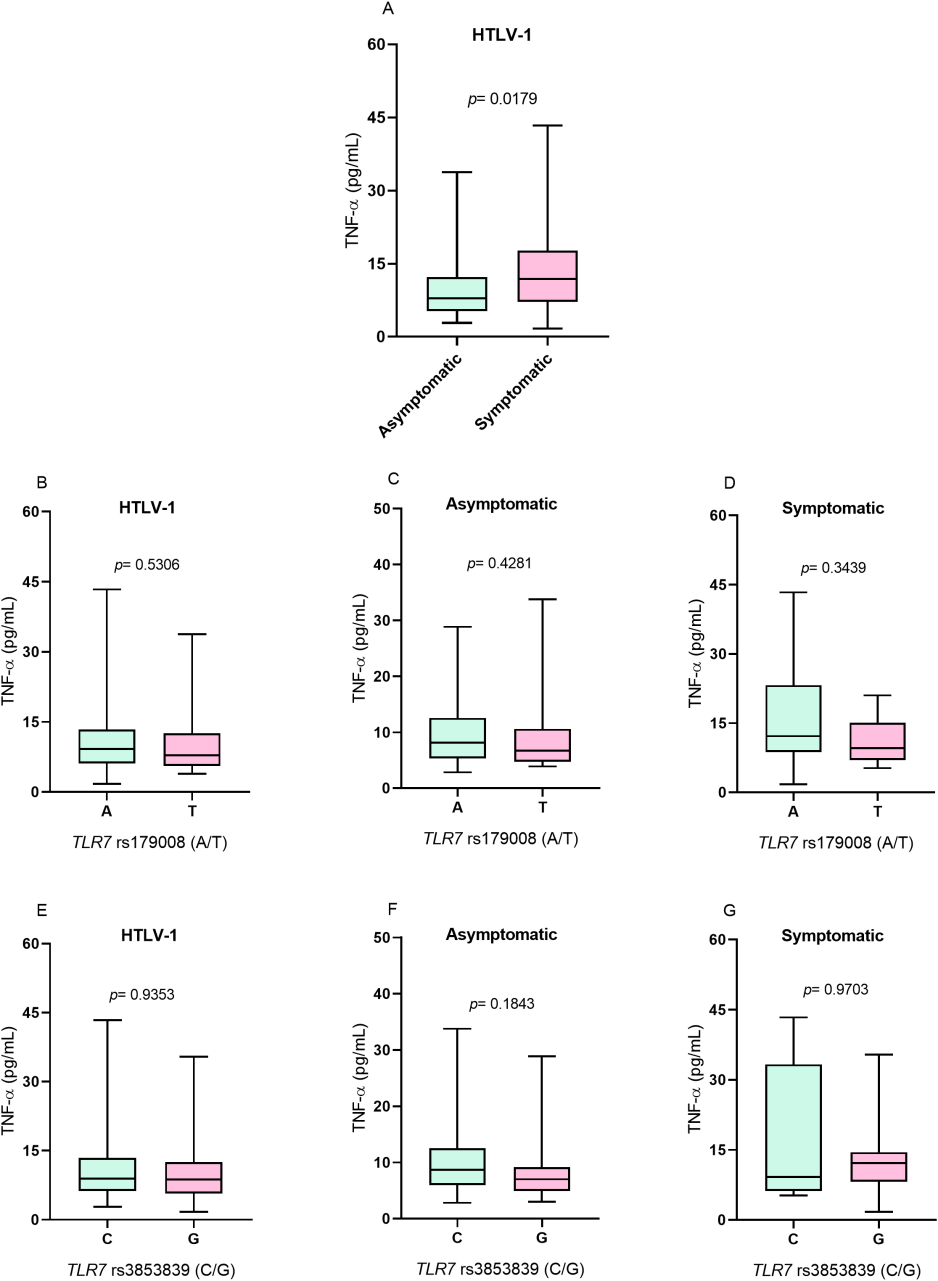
Evaluation of TNF-α levels. **(A)** Asymptomatic individuals and those with symptoms of HTLV-1-associated diseases. **(B)** Individuals living with HTLV-1, **(C)** asymptomatic individuals and **(D)** symptomatic individuals carrying the alleles related and not related to alterations in the protein structure for the *TLR7* rs179008 (A/T) polymorphism. **(E)** Individuals living with HTLV-1, **(F)** asymptomatic individuals and **(G)** symptomatic individuals carrying the alleles related and not related to changes in expression levels for *TLR7* rs3853839 (C/G). Mann-Whitney test. Allele A: includes carriers of the AA (female) and A (male) genotypes; allele T: includes carriers of the AT and TT (female) and T (male) genotypes; allele C: includes carriers of the CC (female) and C (male) genotypes; Allele G: includes carriers of the CG and GG (female) and G (male) genotypes.

The levels of the inflammatory cytokine TNF-α were greater in the symptomatic group than in the asymptomatic group (*p*= 0.0179; **Figure 3A**). However, to *TLR7* rs179008 (A/T) polymorphism for there was no difference in cytokine levels between individuals who were carriers of alleles related (T) and not related (A) to alterations in the structure of the TLR7 protein in individuals with HTLV-1 (**Figure 3B**) or according to the absence (**Figure 3C**) and presence (**Figure 3D**) of symptoms of diseases associated with infection. For *TLR7* rs3853839 (G/C), TNF-α levels were not associated with alleles related (G) and not related (C) to changes in *TLR7* expression levels in individuals with HTLV-1 (**Figure 3E**) or according to the absence (**Figure 3F**) and presence (**Figure 3G**) of symptoms of diseases associated with the virus.

### Quantification of HTLV-1 proviral load

**Figure 4:**
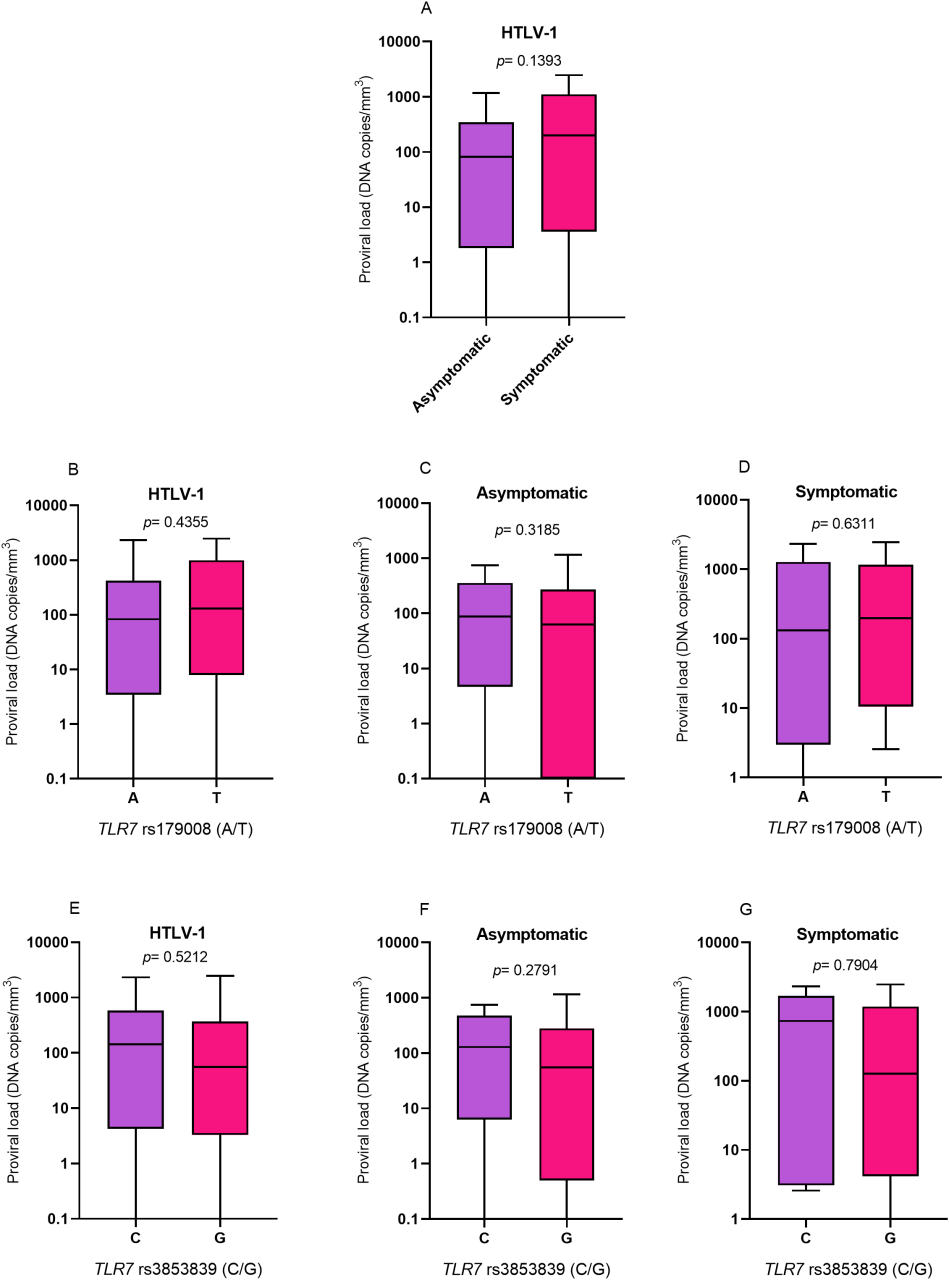
Evaluation of HTLV-1 proviral loads. **(A)** Asymptomatic individuals and those with symptoms of HTLV-1-associated diseases. **(B)** Individuals living with HTLV-1, **(C)** asymptomatic individuals and **(D)** symptomatic individuals carrying the alleles related and not related to alterations in the protein structure for the *TLR7* rs179008 (A/T) polymorphism. **(E)** Individuals living with HTLV-1, **(F)** asymptomatic individuals and **(G)** symptomatic individuals carrying the alleles related and not related to changes in expression levels for *TLR7* rs3853839 (C/G). Mann-Whitney test. Allele A: includes carriers of the AA (female) and A (male) genotypes; allele T: includes carriers of the AT and TT (female) and T (male) genotypes; allele C: includes carriers of the CC (female) and C (male) genotypes; Allele G: includes carriers of the CG and GG (female) and G (male) genotypes.

The proviral loads were evaluated according to the presence and absence of symptoms of diseases associated with HTLV-1 (**Figure 4A**), the alleles related (T) and not related (A) to alterations in the protein structure for the *TLR7* rs179008 polymorphism (A/T) (**Figures 4B-D**) and the alleles related (G) and not related (C) to a change in *TLR7* expression levels for the *TLR7* rs3853839 polymorphism (C/G) (**Figures 4E–G**). However, there were no differences in cytokine levels in any of the analyses.

Evaluation of *TLR7*, IFN-α, TNF- α and proviral load levels in relation to females showed that only carriers of the AA genotype for *TLR*7 rs179008 (A/T) had significantly higher levels of IFN-α (**Supplementary Table S1**).

### Multiple evaluation of variables

Multiple logistic regression was used to evaluate whether the investigated variables could be associated with the presence of symptoms of HTLV-1-related diseases. The analysis showed that only increased proviral load contributed to the outcome of symptoms (*OR*: 0.010; 95% CI: 1,001 to 1,030; *p*= 0.0236) (**Table 5**).

**Table 5:** Multiple logistic regression analysis of the variables investigated in relation to symptoms of HTLV-1-associated diseases.

Variables	*OR*	95% CI	*p*
Symptoms			
*TLR7* rs179008 (A/T) alleles			
A	Ref		
T	1.742	0.389 − 7.796	0.4677
*TLR7* rs3853839 (C/G) alleles			
C	Ref		
G	2.924	0.668 − 12.793	0.1540
*TLR7* expression	1.323	0.888 − 1.969	0.1677
IFN-α	0.995	0.956 − 1.036	0.8258
TNF-α	1.047	0.950 − 1.154	0.3520
Proviral load	1.010	1.001 − 1.030	0.0236

*OR*, *odds ratio*; CI, confidence interval; Ref, reference. Allele A: includes carriers of the AA (female) and A (male) genotypes; allele T: includes carriers of the AT and TT (female) and T (male) genotypes; allele C: includes carriers of the CC (female) and C (male) genotypes; Allele G: includes carriers of the CG and GG (female) and G (male) genotypes.

Multiple linear regression analysis revealed an association between the rs3853839 polymorphism (C/G) and an increase in *TLR7* expression levels (β: 1.281; 95% CI: 0.433 to 2.128; *p*= 0.0036). Regarding IFN-α, the polymorphic allele (T) for the *TLR7* rs179008 (A/T) polymorphism was associated with a decrease in cytokine levels (β: - 1.241; 95% CI: -21.102 to -3.713; *p*= 0.0059). The polymorphic allele (G) for the *TLR7* rs3853839 polymorphism (C/G) was associated with an increase in IFN-α levels (β: 8.718; 95% CI: 0.194 to 17.241; *p*= 0.0452). Increased TNF-α levels were associated with the polymorphic allele (T) for the *TLR7* rs179008 (A/T) polymorphism (β: 5.320; 95% CI: 1.291 to 9.348; *p*= 0.0151) and with increased proviral load (β: 0.005; 95% CI: 0.001 to 0.008; *p*= 0.0017) (**Table 6**).

**Table 6:** Multiple linear regression analysis of *TLR7*, IFN-α and TNF-α gene expression levels.

Variables	Estimate	95% CI	*p*
*TLR7* gene expression			
*TLR7* rs179008 (A/T) alleles			
A	Ref		
T	-0.322	-1.235 − 0.589	0.4818
*TLR7* rs3853839 (C/G) alleles			
C	Ref		
G	1.281	0.433 − 2.128	0.0036
Proviral load	-0.001	-0.005 − 0.001	0.7817
IFN-α			
*TLR7* rs179008 (A/T) alleles			
A	Ref		
T	-1.241	-21.102 − -3.713	0.0059
*TLR*7 rs3853839 (C/G) alleles			
C	Ref		
G	8.718	0.194 − 17.241	0.0451
Proviral load	0.003	-0.003 − 0.006	0.9476
TNF-α			
*TLR7* rs179008 (A/T) alleles			
A	Ref		
T	5.320	1.291 − 9.348	0.0151
*TLR7* rs3853839 (C/G) alleles			
C	Ref		
G	-0.679	-4.422 − 3.063	0.5009
Proviral load	0.005	0.001 − 0.008	0.0017

CI, confidence interval; Ref, reference. Allele A: includes carriers of the AA (female) and A (male) genotypes; allele T: includes carriers of the AT and TT (female) and T (male) genotypes; allele C: includes carriers of the CC (female) and C (male) genotypes; Allele G: includes carriers of the CG and GG (female) and G (male) genotypes.

Based on the results of the multiple linear regression analysis showing a different relationship between IFN-α levels and the polymorphic alleles for the *TLR7* rs179008 (A/T) and *TLR7* rs3853839 (C/G) polymorphisms, IFN-α levels and the other markers were evaluated according to the combination of the following alleles for these polymorphisms: (i) T/C (changes in receptor structure and lower expression levels of *TLR7*), (ii) A/G (preserved receptor structure and increased *TLR7* expression levels), (iii) A/C (preserved receptor structure and decreased *TLR7* expression levels) and (iv) T/G (changes in receptor structure and increased *TLR7* expression levels).

**Figure 5:**
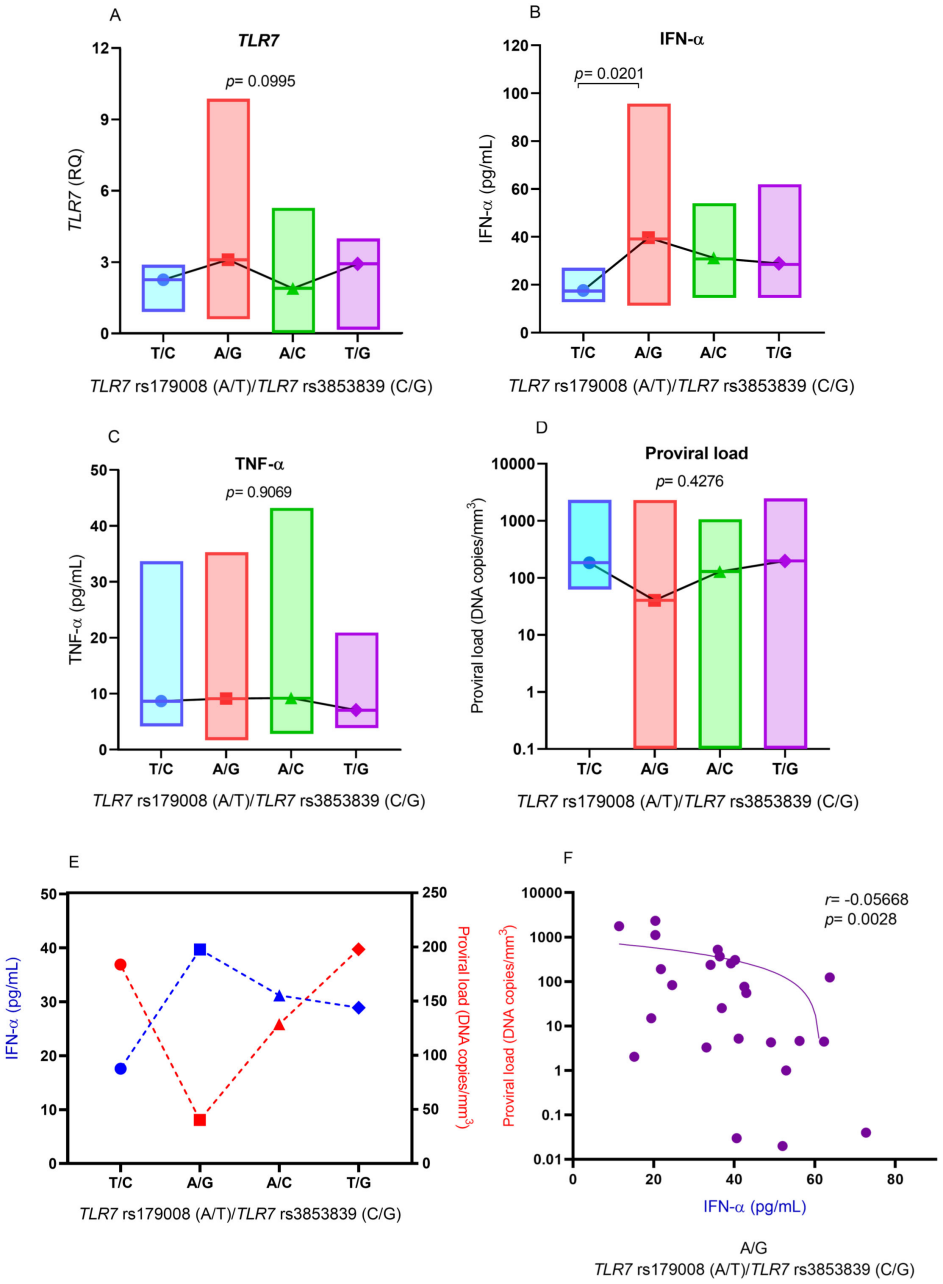
Comparison of the levels of **(A)** IFN-α, **(B)** *TLR7* gene expression, **(C)** TNF-α and **(D)** HTLV-1 proviral load according to the combination of alleles for the *TLR7* rs179008 (A/T) and *TLR7* allele rs3853839 (C/G) polymorphisms. Evaluation of **(E)** median IFN-α levels and proviral loads in relation to the combination of alleles for the polymorphisms; **(F)** correlation of IFN-α levels and proviral loads in individuals with the A/G combination. Kruskal-Wallis test **(A-E)**. Spearman correlation test **(F)**. Allele A: includes carriers of the AA (female) and A (male) genotypes; allele T: includes carriers of the AT and TT (female) and T (male) genotypes; allele C: includes carriers of the CC (female) and C (male) genotypes; Allele G: includes carriers of the CG and GG (female) and G (male) genotypes.

There was no significant difference in *TLR7* expression levels between individuals with different combinations, but individuals with combinations that included the G allele to *TLR7* rs3853839 (C/G) polymorphism had higher *TLR7* expression levels (**Figure 5A**). IFN-α levels were significantly greater in individuals with the A/G allele than in individuals with the T/C allele combination (*p*= 0.0220; **Figure 5B**). The levels of TNF-α (**Figure 5C**) and the proviral load (**Figure 5D**) were not significantly different between individuals carrying the different combinations of alleles for the *TLR7* rs179008 (A/T) and *TLR7* rs3853839 (C/G) polymorphisms. However, carriers of the A/G alleles had lower HTLV-1 proviral loads (**Figure 5D**). The comparison of the medians of IFN-α levels and the viral loads of patients with HTLV-1 was influenced by the combination of the A/G alleles. Individuals carrying these alleles had higher levels of IFN-α and lower proviral loads (**Figures 5E, F**).

## Discussion

TLR7 recognizes ssRNA and induces the production of large amounts of IFN-α and inflammatory cytokines, which are responsible for promoting the control of viral infections (34). Variations in *TLR7* expression levels may influence the development of chronic diseases caused by HBV infection (35), the process of fibrosis induced by HCV infection (36), HIV-1 viremia (37) and induce the pathogenesis of SARS-CoV-2 (38).

Initially, the present study revealed an association of the polymorphic allele of the *TLR7* rs179008 (A/T) polymorphism with the presence of symptoms of HTLV-1-associated diseases. However, multiple logistic regression analysis revealed that the *TLR7* rs179008 polymorphism (A/T) and the *TLR7* rs3853839 variation (C/G) were not associated with clinical manifestations resulting from viral infection. On the other hand, both bivariate analysis and multiple linear regression showed an association of the *TLR7* rs179008 (A/T) polymorphism with reduced levels of IFN-α and of the polymorphic allele for the *TLR7* rs3853839 genetic variation (C/G) with increased *TLR7* gene expression levels.

The *TLR7* rs179008 (A/T) polymorphism was associated with a worse prognosis during the course of HIV-1 infection; individuals with this polymorphism had greater viral loads and faster progression to immunosuppression (23). In these individuals, the *TLR7* rs179008 (A/T) polymorphism decreased the production of IFN-α (23). Women with acute HIV-1 infection carrying the *TLR7* rs179008 (A/T) polymorphism had reduced viral load and lower frequency of clinical symptoms (24). In SARS-CoV-2 infection, the *TLR7* rs179008 (A/T) polymorphism was associated with the severity of COVID-19 (26). Regarding the *TLR7* rs3853839 (C/G) variation, the polymorphic allele was associated with a greater likelihood of individuals developing COVID-19, with disease severity and poor clinical parameters, including hospitalization, respiratory failure, cardiac complications, intensive care unit hospitalization and mechanical ventilation (30). In enterovirus 71 (EV71) infection, the G allele was associated with a lower risk of serious diseases related to higher *TLR7* gene expression and IFN-α levels (39). Although individuals with HCV infection carrying the *TLR7* rs3853839 (C/G) polymorphism exhibited higher *TLR7* gene expression and IFN-α levels than did those with the wild-type genotype, the frequency of the wild-type genotype was greater in the group with persistent infection (29).

These results show the relevance of the *TLR7* rs179008 (A/T) and *TLR7* rs3853839 (C/G) polymorphisms in the context of viral infections, as these polymorphisms seem to contribute to the evolution of infections caused by different viruses, including HTLV-1. However, it is important to note that the association of polymorphisms may be influenced by other factors inherent to the type of infection (acute or chronic), the biology of the virus and the immunological condition of the individual during the infection process because, as we observed in the findings of previous studies, the polymorphisms may be associated with different clinical outcomes. Although, in our study, the polymorphisms were related to changes in the levels of *TLR7* expression and IFN-α production in HTLV-1 infection, did not directly influence the induction of disease symptoms, while the relationship of these polymorphisms with cases of serious illness is much stronger in other infections.

In our study, the HTLV-1 proviral load was greater in individuals with symptoms of disease than in asymptomatic individuals, although this difference was not statistically significant. However, the proviral load was not associated with the *TLR7* rs179008 (A/T) or *TLR7* rs3853839 (C/G) polymorphisms. The quantification of the proviral load is considered an important parameter for the evaluation of HTLV-1 infection (40). A greater proviral load is associated with the presence of disease symptoms, especially in HAM (41, 42), although some individuals without clinical symptoms of specific diseases associated with HTLV-1 may also present high proviral loads (40), suggesting that these individuals need constant medical monitoring to assess the onset of symptoms earlier. As HTLV-1 is detected by TLR7, the *TLR7* rs3853839 (C/G) and *TLR7* rs179008 (A/T) polymorphisms do not appear to be sufficient to influence antiviral activity and control virus replication when present in isolation.

Elevated TNF-α level were associated with the presence of symptoms of HTLV-1-related diseases in the bivariate analysis; however, this was not confirmed in the multivariate analysis. Although HTLV-1 induces inflammatory diseases such as HAM, which is associated with increased TNF-α levels (43), it is possible that this cytokine is more concentrated at the site of the inflammatory process, with lower detection at the systemic level. Other factors inherent to the individual’s physiological condition may contribute to variations in cytokine levels, such as obesity, since adipose tissue induces the production of TNF-α (44).

Multiple regression analysis revealed that the *TLR7* rs3853839 (C/G) polymorphism was associated with increased *TLR7* gene expression levels and increased IFN-α levels and that the *TLR7* rs179008 (A/T) polymorphism was associated with reduced INF-α levels. The levels of the investigated markers were evaluated in relation to different combinations of alleles for the *TLR7* rs3853839 (C/G) and *TLR7* rs179008 (A/T) polymorphisms. In this analysis, IFN-α levels were significantly greater in individuals carrying the A/G allele, which is related to the preserved structure of the TLR7 receptor and higher *TLR7* gene expression levels for the *TLR7* rs179008 (A/T) and *TLR7* rs3853839 polymorphisms (C/G), respectively. These same individuals also had lower HTLV-1 proviral loads, although the differences were not statistically significant. These results suggest an important relationship between the alleles that influence the gene expression and structure of TLR7 and HTLV-1 infection. Combinations of different alleles for variations in the *TLR7* gene were associated with the evolution of viral infections. The combination of TTA alleles for the *TLR7* polymorphisms rs179010 (C/T), rs2074109 (T/C) and rs179009 (A/G) was associated with reduced susceptibility to HIV-1 infection (17), while the CTA haplotype was associated with the progression of chronic hepatitis B (45). Our study showed that although the *TLR7* rs179008 (A/T) and *TLR7* rs3853839 (C/G) polymorphisms do not allow differentiating the presence or absence of symptoms of HTLV-1-associated diseases, it is possible that the combination of A/G alleles to these polymorphisms may influence the antiviral response to the course of HTLV-1 infection.

Our study presents important information that may contribute to the context of personalized medicine, related to the prediction of prognosis of HTLV-1 infection, since mutations in TLR7 can influence the evolution of viral infections. However, the study has some limitations, including the number of polymorphisms evaluated. The study evaluated two polymorphisms in the TLR7 gene, but there are other genetic variations that need to be investigated in order to identify which ones may be contributing to better or worse control of HTLV-1 infection. Another limitation is related to cytokine levels. Several factors can influence systemic cytokine levels. The presence of infection is one of these main factors, although some conditions can contribute to variations in cytokine levels, such as obesity, age and smoking. Although HTLV-1 infection was present in both groups (symptomatic and asymptomatic) and the mean age of these groups was similar, individuals were not assessed for obesity and smoking habits. In summary, the *TLR7* rs179008 (A/T) polymorphism was associated with reduced IFN-α levels, and the *TLR7* rs3853839 (C/G) variation was associated with increased TLR7 gene expression and IFN-α levels. The polymorphisms were not associated with the presence of symptoms of HTLV-1-associated diseases; however, in combination, the A and G alleles for the TLR7 rs179008 (A/T) and TLR7 rs3853839 (C/G) polymorphisms appear to be important in controlling HTLV-1 infection, as they have been shown to promote a greater antiviral response against HTLV-1 infection.

## Data Availability

The original contributions presented in the study are included in the article/**Supplementary Material**. Further inquiries can be directed to the corresponding author.
