# Supplementary material for: TLR7 rs179008 (A/T) and TLR7 rs3853839 (C/G) polymorphisms are associated with variations in IFN-α levels in HTLV-1 infection
Source: Front Immunol. 2024 Nov 22;15:1462352. doi: 10.3389/fimmu.2024.1462352 (PMC11621001; doi:10.3389/fimmu.2024.1462352)
Supplement: Supplementary file 1 [file Table1.docx]

**Supplementary Table 1** - Comparison of median levels of TLR7 gene expression, cytokines and proviral load of female individuals carrying different genotypes for TLR7 rs179008 (A/T) according to the presence and absence of symptoms of HTLV-1-associated diseases.

| **Genotypic profile** | **TLR7 (RQ)** Median/IQR | **IFN-A (pg/mL)** Median/IQR | **TNF-A** **(pg/mL)** Median/IQR | **PVL (cell/uL)** Median/IQR |
| --- | --- | --- | --- | --- |
| **Asymptomatic** |  |  |  |  |
| AA | 2.537/2.590 | 35.3/11.8 | 7.60/4.80 | 76.60/156.72 |
| AT | 2.421/0.642 | 26.5/9.1 | 11.96/2.67 | 74.98/183.78 |
| TT | 0.142/0.884 | 20.9/5.2 | 10.24/12.28 | 127.5/625.49 |
| *p** | 0.1095 | 0.0127 | 0.1347 | 0.9581 |
|  |  |  |  |  |
| **Symptomatic** |  |  |  |  |
| AA | 2.737/2.358 | 34.1/19.1 | 11.88/3.68 | 25.28/1108.19 |
| AT | 2.931/0.994 | 21.8/18.6 | 8.56/9.81 | 197.9/706.49 |
| TT | 3.488/0.754 | 17.6/2.1 | 17.66/0.34 | 9.67/2.01 |
| *p** | 0.8488 | 0.3384 | 0.6533 | 0.7751 |

IQR: interquartile range; *Kruskal-Wallis test.
